# A *ν*-support vector regression based approach for predicting imputation quality

**DOI:** 10.1186/1753-6561-6-S7-S3

**Published:** 2012-11-13

**Authors:** Yi-Hung Huang, John P Rice, Scott F Saccone, José Luis Ambite, Yigal Arens, Jay A Tischfield, Chun-Nan Hsu

**Affiliations:** 1 Institute of Information Science, Academia Sinica, Taipei 115, Taiwan; 2 Department of Computer Science, National Taiwan University, Taipei 106, Taiwan; 3 Department of Psychiatry, Washington University, St. Louis, Missouri, USA; 4 Information Science Institute, University of Southern California, Marina del Rey, California, USA; 5 Department of Genetics, Rutgers University, Piscataway, New Jersey, USA

## Abstract

**Background:**

Decades of genome-wide association studies (GWAS) have accumulated large volumes of genomic data that can potentially be reused to increase statistical power of new studies, but different genotyping platforms with different marker sets have been used as biotechnology has evolved, preventing pooling and comparability of old and new data. For example, to pool together data collected by 550K chips with newer data collected by 900K chips, we will need to impute missing loci. Many imputation algorithms have been developed, but the posteriori probabilities estimated by those algorithms are not a reliable measure the quality of the imputation. Recently, many studies have used an imputation quality score (IQS) to measure the quality of imputation. The IQS requires to know true alleles to estimate. Only when the population and the imputation loci are identical can we reuse the estimated IQS when the true alleles are unknown.

**Methods:**

Here, we present a regression model to estimate IQS that learns from imputation of loci with known alleles. We designed a small set of features, such as minor allele frequencies, distance to the nearest known cross-over hotspot, *etc*., for the prediction of IQS. We evaluated our regression models by estimating IQS of imputations by BEAGLE for a set of GWAS data from the NCBI GEO database collected from samples from different ethnic populations.

**Results:**

We construct a *ν*-SVR based approach as our regression model. Our evaluation shows that this regression model can accomplish mean square errors of less than 0.02 and a correlation coefficient close to 0.75 in different imputation scenarios. We also show how the regression results can help remove false positives in association studies.

**Conclusion:**

Reliable estimation of IQS will facilitate integration and reuse of existing genomic data for meta-analysis and secondary analysis. Experiments show that it is possible to use a small number of features to regress the IQS by learning from different training examples of imputation and IQS pairs.

## Background

In the past decade, the data sets collected for genome wide association studies (GWAS) have grown geometrically. Reusing these valuable data in new studies is difficult because they are collected through different study designs and on different platforms. Various imputation algorithms (*e.g.*, IMPUTE [1], BEAGLE [2-7], and MACH [8]) have been developed to predict the individual genotypes at un-typed markers. Although these imputation algorithms have already been put to use, the methods of measuring imputation quality are still rarely addressed. The imputation quality score of the single-nucleotide polymorphism (SNP) genotypes are quite different at distinct loci. For this reason, we want to investigate how to measure the imputation quality for a particular SNP that is imputed by these algorithms. After the imputation quality measurement is established, researchers can pay more attention to those poorly-imputed SNPs in the data integration process. Recently, [9] proposed a new statistic for assessing the imputation reliability and it is designated as the imputation quality score (IQS). The IQS has been shown to be commensurate with the true quality of the imputation and successfully applied to filter false positive associations in GWAS studies that use imputed genotypes [9].

The IQS for each imputed SNP is computed by two scores, the proportion of observed agreement (*P_o_*) and the proportion of chance agreement (*P_c_*), to account not just for the accuracy of the imputation but also whether it is accurate by chance alone. In detail, the computation of IQS requires the posterior probabilities of AA, AB and BB as output by the imputation program. For one SNP genotyped on *N *individuals, the probabilities can be readily constructed as shown in Table 1 where each cell, *n_ij_*, represents the number of individuals with true genotype *j *and imputed genotype *i*. The observed agreement *P_o _*is defined in percentage $P_{o}=\frac{\sum_{i}n_{ii}}{n_{\cdot \cdot }}$. Similar to *P_o_*, The chance agreement *P_c _*is defined as the proportion of agreement which is expected by chance: $P_{c}=\frac{\sum_{i}{n_{i}}_{\cdot }{n_{\cdot }}_{i}}{{n_{\cdot \cdot }}^{2}}$, where *n_i._*, *n_.i_*, and *n*_.. _are defined in Table 1. Then IQS is calculated by the Cohen's kappa coefficient [10] and is defined as a function of *P_o _*and *P_c _*as

**Table 1:** Marginal cross classification of the genotypes used for the computation of IQS

True genotypes				
**Imputed Genotypes**	***AA***	***AB***	***BB***	***Total***

*AA*	*n*_11_	*n*_12_	n_13_	*n*_1._
*AB*	*n*_21_	*n*_22_	*n*_23_	*n*_2._
*BB*	*n*_31_	*n*_32_	*n*_33_	*n*_3._
*Total*	*n*_.1_	*n*_.2_	*n*._3_	*n*..

$$IQS=\frac{P_{o}-P_{c}}{1-P_{c}}.$$

Assessment of *P_o_*, *P_c_*, and IQS needs the true genotypes to be known. [9] showed that for the same population and the same locus imputed using the same set of loci with known genotypes, the estimated IQS are highly correlated. We showed it by dividing a sample by half and imputing SNPs of the Illumina 1 M array using the SNP genotyping results from the Illumina 550 K array, and then we estimated the IQS scores. We obtained a correlation coefficient of 0.99 for the IQS scores for the same set of imputed SNPs. That is, we can expect that IQS scores will be nearly the same if the population, the imputed SNPs, and the SNPs of known genotypes, are identical. If there are previously estimated IQS scores available that match these conditions, then the scores can be reused. Therefore, it is possible to obtain IQS scores without knowing true genotypes by querying the IQS from a pre-constructed IQS database.

However, exhausting all populations and combinations of imputation loci to establish such a database of all useful IQS may take considerable resources. Here, we try to develop a computational method to estimate IQS without known true genotypes. We assess whether or not it is possible to build a regression model from imputations of SNP sites with known alleles, and then use the regression model to estimate IQS for SNPs with unknown alleles. The idea is to use additional statistical information to build a regression model to predict the IQS. Also, in practice, people work with specific sets of variants and this method will facilitate creation of a database of the IQS of those variants.

## Methods and materials

### *ν***-Support vector regression**

In a multi-dimensional regression problem, we have a data set of *l d*-dimensional independent variables *x_i _*∈ ℝ*^d^*, *i *= 1,..., *l *and dependent variables *y_i _*∈ ℝ. In our IQS regression problem, *y_i _*represents the true IQS and *x_i _*denotes the input feature vector. The goal is to find a function that approximates *y_i_*. A solution of this problem based on a kernel method is to find the function *y_i _*≈ *f*(*x_i_*, *w*, *b*) = *w*. *φ *(*x_i_*) - *b*, where *w *and *b *∈ ℝ*^d ^*are parameters and *φ : *ℝ*^d ^*→ ℝ*^d ^*is a mapping such that there exists a kernel function that computes the inner product *φ *(*x_i_*). *φ *(*x_j_*) = *k*(*x_i_*, *x_j_*). Because the radial basis function (RBF) can preserve a relatively high accuracy in comparison with other kernel functions (data not shown), our choice of the kernel function is the RBF kernel [11,12].

Many models and algorithms have been developed to search for the parameters *w *and *b *of the regression function that maximally ts the input set of data. The *ε*-Support Vector Regression model (*ε*-SVR) is one of the useful models. Its formulation is given as:

(1) $$\begin{array}{c} minimize\;\frac{1}{2}\;||w|{|}^{2}+C\overset{l}{\underset{i=1}{\sum }}\left(\xi_{i}+\xi_{i}^{*}\right)\\ subject\;to\;\left\{\begin{array}{c} y_{i}-w\cdot \varphi \left(x_{i}\right)-b\leq \varepsilon +\xi_{i}\\ w\cdot \varphi \left(x_{i}\right)+b-y_{i}\leq \varepsilon +\xi_{i}^{*}\\ \xi_{i},\xi_{i}^{*}\geq 0. \end{array}\right) \end{array}$$

The parameter *C *is used to determine the complexity of model and controls the tradeoff between the training error minimization and the model complexity. If it is too small, the model may underfit the data. The parameter *ε *serves as the tolerance of errors of the regression. Combined with the slack variables ξ*_i _*$\xi_{i}^{*}$ we have a soft-margin approach to regression that can be flexibly adjusted [11,12].

The *ν*-Support Vector Regression (*ν*-SVR) introduces another parameter *ν *in the formulation, which is proven to be easier to adjust than *C*. One of the reasons is that the range of *ν *is [0,1] while the range of *C *is [0, ∞) [13-15].

(2) $$\begin{array}{c} minimize\;\frac{1}{2}||w|{|}^{2}+C\left(v\varepsilon +\frac{1}{l}\overset{l}{\underset{i=1}{\sum }}\left(\xi_{i}+\xi_{i}^{*}\right)\right)\\ subject\;to\;\left\{\begin{array}{c} y_{i}-w\cdot \varphi \left(x_{i}\right)-b\leq \varepsilon +\xi_{i}\\ w\cdot \varphi \left(x_{i}\right)+b-y_{i}\leq \varepsilon +\xi_{i}^{*}\\ \xi_{i},\xi_{i}^{*}\geq 0,\varepsilon \geq 0 \end{array}\right) \end{array}$$

Moreover, the parameter *ν *can serve as an upper bound for the fraction of margin errors, and a lower bound for the fraction of the number of support vectors. In comparison with *C*, to select a suitable *ν *would be more intuitive [13,14]. Therefore, we chose *ν*-SVR over *ε*-SVR for our IQS prediction model. This model is also known to provide high out-of-sample generalization performance.

We chose LibSVM [16] as our implementation of the *ν*-SVR model. The parameter *γ *in the radial basis function is set as 1/*d*. The parameter *ν *was searched within {0.1, 0.2, 0.3, . . . 1.0} and an optimal value of *ν *were selected by applying a 10-fold cross validation on the training data set. The regression model can be applied to approximate *P_o _*and *P_c _*as well as the IQS.

### Features generation

Other regression models can also be used but the key to the success is to identify a set of variables that influence the imputation quality as the input features *x_i _*in the regression model. We intended to use all useful information related to imputation quality as features for the regression model. Under consideration of the statistical correlation analysis (data not shown), we selected the following 12 defined features of a SNP whose allele we want to impute within a given sample.

1. Chromosome position: The chromosome where the SNP located.

2. Physical position: The position of the imputed SNP in bp.

3. Minor allele frequency (MAF): Previously, [9] have shown that the minor allele frequency is an important variable correlated with the true IQS. The above three features are available in the annotation file from the genotyping platform provider.

4. B allele frequency: This is derived from the allele signal intensity measurement for each locus of each individual in the raw CEL files. The raw CEL files are available from the Hapmap samples [17]. For each imputed SNP, we used the mean of the B allele frequency of the SNP on the samples of the corresponding ethnic population.

5. MAF in the reference panel: In addition to using the available MAF provided by the annotation file, we also consider the MAF in the reference panel.

6. Ratio of genotypes AA/AB: It is used to to indicate the proportion of genotype AA for each imputed SNP in the reference panel.

7. Ratio of genotypes BB/AB: Similar to feature 6, it is used to to indicate the proportion of genotype BB for each imputed SNP in the reference panel.

8. Distance to the nearest genotyped SNP: This is to capture an indication that the imputation quality will be better if the nearest genotyped SNP in the inference panel is closer.

9. Distance to the nearest recombination hotspot: The distance to the nearest recombination hotspot also plays an important role in the quality of the imputation. We used the recombination rates and hotspots available in the release version phase II build b35 to GRCh37 from the International HapMap Project [17].

10. The nearest recombination hotspot's recombination rate (cM/Mb, centiMorgans per megabase): This variable is important in the imputation process. The IMPUTE2 program uses it explicitly as a required input for the imputation [1,18].

11. Posterior probability estimated by the imputation program: This variable is available from the output of the imputation program. The Beagle program provides the genotype probabilities file and the genotype dosage file. We used the mean values of the posterior probabilities estimated for all the individuals in the inference panel.

12. B-allele dosage: Given the posterior genotype probabilities for a SNP (Pr(*AA*), Pr(*AB*), and Pr(*BB*)), the estimated B-allele dosage for each individual is equal to 0 × Pr(*AA*) + 1 × Pr(*AB*) + 2 × Pr(*BB*), which is reported in the genotype dosage file. We used the mean values of the B-allele dosage values of all the individuals in the inference panel.

It is worth mentioning that the posterior probability estimated by the imputation program and the B-allele dosage are highly correlated to predicting the IQS under the statistical correlation analysis. These features will be used in the regression model for the IQS as well as the regression for the observed agreement *P_o _*and the chance agreement *P_c_*. We will show that these 12 features are useful to construct an adequate regression model.

### Data preparation

We prepared three data sets to evaluate the performance of our regression models. These data sets contain genotyping results of samples chosen to cover different ethnic backgrounds collected in different disease studies. We selected recent data sets genotyped with advanced platforms that cover a large number of SNPs so that we can flexibly keep those SNPs covered by old, obsolete platforms (with less SNPs probed) and hold out the rest to impute. Meanwhile, since we have their true genotypes, we can use the true genotypes of these SNPs as the gold standard to evaluate imputation quality and regression.

The Merlion Lung Cancer Study 2 DNA [19] and Oral Squamous Cell Carcinoma samples [20] from the NCBI GEO database [21] were used for evaluating the regression model. The Merlion Lung Cancer samples consist of two ethnic populations, East-Asian (EA) and Western-European (WE). Samples were all genotyped on the Affymetrix Genome-Wide Human SNP Array 6.0 platform. This platform contains more than 906,600 SNP probes, including the historical 482,000 SNPs in the Affymetrix GeneChip Human Mapping 500K Array Set. After the preprocessing of raw CEL files, there are 763,252 SNPs reported for the EA population of Merlion Lung Cancer samples, 778,058 SNPs for the WE population of Merlion Lung Cancer samples, and 693,494 SNPs for the oral Squamous Cell Carcinoma samples.

### Regression performance evaluation

We designed scenarios to simulate the imputation of missing SNPs in a data set genotyped using an old platform to the large set of SNPs on the Affymetrix SNP 6.0 array. These scenarios involve a *training set *to construct our regression model in advance. This involves holding out a set of SNPs to impute, evaluating true IQS with known alleles, using the true IQS to train the regression model. Then the trained regression model can be applied to estimate IQS of imputed SNPs in a *test set*, where a set of SNPs is assumed to have missing genotypes. The design of the scenarios is to create different combinations of the training and test sets and see how the regression performance is affected.

To create both training and test sets, we basically divided the SNPs on the Affymetrix SNP 6.0 array into two sets. One contains those SNPs genotyped in both an old platform and Affymetrix SNP 6.0 array. This set simulates SNPs with "known" genotypes to be used to impute other SNPs. The other contains the remaining SNPs covered only by the Affymetrix SNP 6.0 array. This set simulates "missing" SNPs to be imputed.

Table 2 and Table 3 show our design of training and test sets for four scenarios to evaluate generalization of the regression model. Scenario 1 is the simplest case, which tests the regression performance when a sample of the same ethnic and disease phenotype is used for training. We used the WE lung cancer sample to create the training set. Alleles of the randomly picked 10% of SNPs of the training set were erased, denoted as "missing." Under the Affymetrix 500k array, these "missing" SNPs were imputed using the other 90% genotyped SNPs to a full set of SNPs on the same platform. As a result, there are 41,304 SNPs of the WE lung cancer sample used for the model training. We also used the WE lung cancer sample to create the test set, which consists of 320,172 SNPs covered only by the Affymetrix SNP 6.0 array. Their genotypes were then imputed from SNPs covered by the Affymetrix mapping 500k array and our regression model was applied to assess the imputation quality.

**Table 2:** Summary of training set composition for different evaluation scenarios

Scenarios	Ethnic population	Samples	from Platform	to Platform
Scenario 1	Western European	Lung cancer	from Affymetrix 500k	to Affymetrix 500k
Scenario 2	Western European	Lung cancer	from Illumina 550k	to Illumina 550k
Scenario 3	East Asian	Lung cancer	from Affymetrix 500k	to Affymetrix 500k
Scenario 4	East Asian	Lung cancer	from Affymetrix 500k	to Affymetrix 500k

**Table 3:** Summary of test set composition for different evaluation scenarios

Scenarios	Ethnic population	Samples	from Platform	to Platform
Scenario 1	Western European	Lung cancer	from Affymetrix 500k	**to Affymetrix SNP 6.0**

Scenario 2	Western European	Lung cancer	**from Affymetrix 500k**	**to Affymetrix SNP 6.0**

Scenario 3	**Western European**	Lung cancer	from Affymetrix 500k	**to Affymetrix SNP 6.0**

Scenario 4	East Asian	**Oral cancer**	from Affymetrix 500k	**to Affymetrix SNP 6.0**

High-lighted fields are the settings that are different from the training set used in the corresponding scenarios.

In Scenario 2, the generalization performance of our IQS regression model was evaluated when it was trained using "known" and "missing" SNPs covered by platforms different from those to be used in testing. We used the WE lung cancer sample again but used the Illumina 550k array instead of the Affymetrix SNP 6.0 array to choose SNPs. There are 41,304 SNPs of the WE lung cancer sample on the Illumina 550k array. After the regression model is constructed, we then used the same test set created in Scenario 1.

In Scenario 3, our IQS regression model is applied to different ethnic populations. We used the EA lung cancer sample to create the training set, resulting in 37,611 SNPs of the EA lung cancer sample on the Affymetrix 500k array. The regression model constructed by the EA lung cancer samples was used to predict the IQS of SNPs of the WE lung cancer samples as in Scenario 1.

Scenario 4 tests if our regression model can be generalized across samples collected for different disease studies. We used the same training set as in the scenarios above and used the EA Oral Squamous Cell Carcinoma sample as the test set. This test set also simulates imputation from the Affymetrix mapping 500k array to the Affymetrix SNP 6.0 array and consists of 320,172 SNPs.

For all scenarios, we chose the imputation program Beagle. Beagle is based on the Hidden Markov Model (HMM) [22]. To estimate missing alleles, an EM algorithm is adopted to optimize the parameters to fit the HMM model from a given genotyped reference panel [2,7]. In terms of imputation accuracy, Beagle perform as well as other imputation programs but is known to be more efficient with regard to running time and memory space required [23].

The 1000 Genomes Project samples (August 2010 release) served as the reference panel. As the larger reference panel has developed, researchers have become more confident to combine two studies or extend a specific study on different platforms [23]. We removed those SNPs with MAF less than 1% that usually lead to decreased imputation accuracy [9,23]. About 2% of SNPs were removed before the imputation. Notably, there are a few SNPs with inconsistent genotyped markers compared to the reference panel. These few SNPs (< 0.01%) will be excluded from the training or test set in order to focus only on the reasonable imputation results.

## Results and discussion

**Figure 1:**
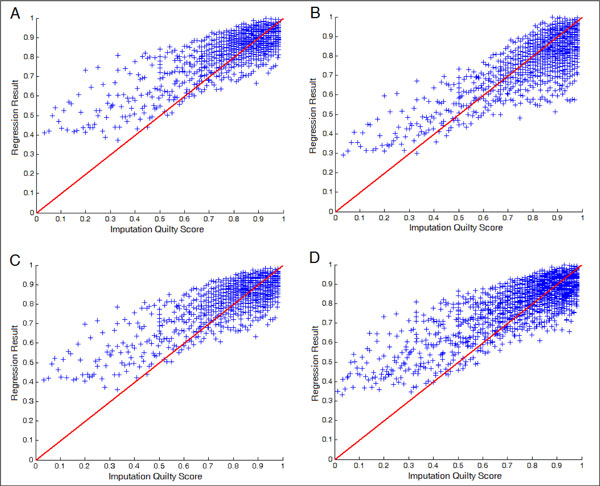
**IQS regression results, **(A) Scenario 1, evaluating the regression result on the same platform. (B) Scenario 2, evaluating the regression result on different platforms. (C) Scenario 3, evaluating the regression result on the different ethnic population. (D) Scenario 4, under the same ethnic population, evaluating the regression result on the different disease samples.

Table 4 shows the regression performance of our model for predicting the IQS under different model training and imputation scenarios and Figure 1 shows the scatter plot. The results show that our regression model achieved mean square errors less than 0.02 and correlation coefficients close to 0.75. The performance is consistent across different scenarios, suggesting that the regression model generalizes equally well in different scenarios. However, Figure 1 shows that regression value usually overestimated values, especially for low IQS imputations.

**Table 4:** Summary of the IQS regression results for each scenario

IQS regression results		
**Scenario**	**Mean Squared Error**	**Correlation Coefficient**

Scenario 1	0.0182	0.740

Scenario 2	0.0174	0.748

Scenario 3	0.0178	0.736

Scenario 4	0.0197	0.751

The best performance was accomplished in Scenario 2, where the regression model was trained with a set of SNPs derived from different platforms from the test, suggesting that training with a wider variety of SNPs might allow the model to generalize better. The worst performance was from Scenario 4, where samples from studies of different diseases were tested. Nevertheless, the performance difference was not significant.

**Figure 2:**
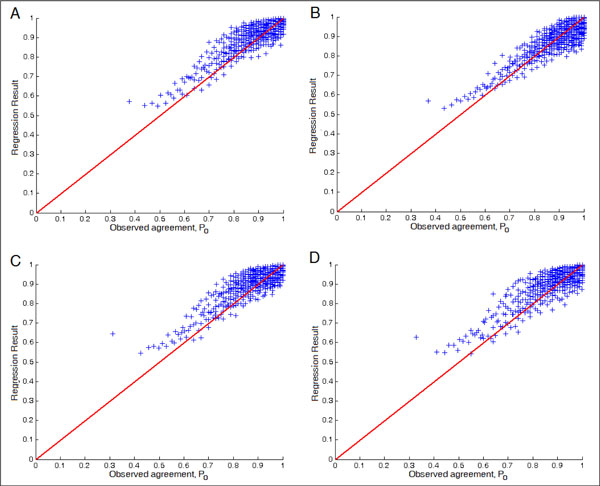
***P**_**o**_***regression results**, (A) Scenario 1, evaluating the regression result on the same platform. (B) Scenario 2, evaluating the regression result on different platform. (C) Scenario 3, evaluating the regression result on the different ethnic population. (D) Scenario 4, under the same ethnic population, evaluating the regression result on the different disease samples.

**Figure 3:**
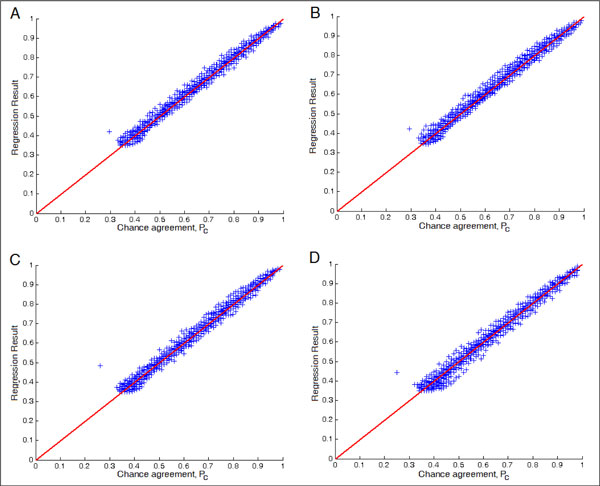
***P**_**c**_***regression results**, (A) Scenario 1, evaluating the regression result on the same platform. (B) Scenario 2, evaluating the regression result on different platform. (C) Scenario 3, evaluating the regression result on the different ethnic population. (D) Scenario 4, under the same ethnic population, evaluating the regression result on the different disease samples.

Tables 5, 6 and Figures 2, 3 show the regression results for *P_o _*and *P_c_*, respectively. It turned out that the results are better than those for the regression of the IQS. The result for *P_c _*is particularly good because *P_c _*is just the marginals. One may speculate that it may be useful to predict *P_o _*and *P_c _*separately and then combine them to obtain the estimated IQS. We tried this approach but the results were similar to directly predicting the IQS.

**Table 5:** Summary of the *P_o _*regression results for each scenario

	*P_o _*regression results	
**Scenario**	**Mean Squared Error**	**Correlation Coefficient**

Scenario 1	0.00248	0.840
Scenario 2	0.00249	0.838
Scenario 3	0.00256	0.835
Scenario 4	0.00301	0.831

**Table 6:** Summary of the *P_c _*regression results for each scenario

	*P_c _*regression results	
**Scenario**	**Mean Squared Error**	**Correlation Coefficient**

Scenario 1	0.00062	0.990
Scenario 2	0.00072	0.988
Scenario 3	0.00071	0.989
Scenario 4	0.00099	0.984

**Figure 4:**
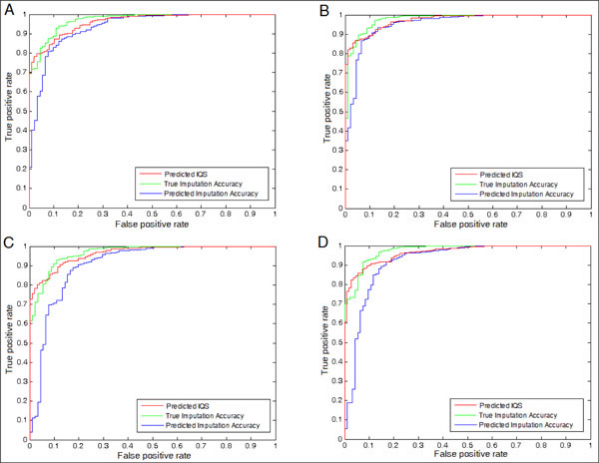
ROC curve at the threshold = 0.5, (A) Scenario 1, AUC(Predicted IQS):0.9617, AUC(True Imputation Accuracy):0.9718, and AUC(Predicted Imputation Accuracy):0.9354 (B) Scenario 2, AUC(Predicted IQS):0.9739, AUC(True Imputation Accuracy):0.9783, and AUC(Predicted Imputation Accuracy):0.9539 (C) Scenario 3, evaluating the regression result on the different ethnic population, AUC(Predicted IQS):0.9642, AUC(True Imputation Accuracy):0.9677, and AUC(Predicted Imputation Accuracy):0.9072 (D) Scenario 4, AUC(Predicted IQS):0.9656, AUC(True Imputation Accuracy):0.9758, and AUC(Predicted Imputation Accuracy):0.9223

**Figure 5:**
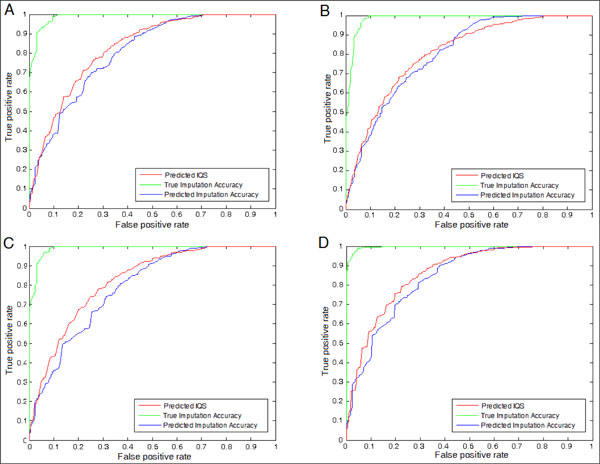
ROC curve at the threshold = 0.9, (A) Scenario 1, AUC(Predicted IQS):0.8269, AUC(True Imputation Accuracy):0.9883, and AUC(Predicted Imputation Accuracy):0.8041 (B) Scenario 2, AUC(Predicted IQS):0.8082, AUC(True Imputation Accuracy):0.9848, and AUC(Predicted Imputation Accuracy):0.8030 (C) Scenario 3, AUC(Predicted IQS):0.8230, AUC(True Imputation Accuracy):0.9892, and AUC(Predicted Imputation Accuracy):0.7890 (D) Scenario 4, AUC(Predicted IQS):0.8620, AUC(True Imputation Accuracy):0.9967, and AUC(Predicted Imputation Accuracy):0.8399

We also performed a test to evaluate whether we can use the regression results to filter out false positives in a GWAS. Previously, [9] showed that by setting a suitable threshold for the true IQS a better filtering rate can be accomplished than by using the imputation accuracy, which is equivalent to *P_o_*. In this test, we assumed that an imputation with a true IQS below a certain threshold can be considered as a true flase positive that must be filtered out. Under this assumption, we plotted the Receiver Operating Characteristic (ROC) curve of the regression results against the presumed false positives. The results are presented as Figure 4 and 5. The predicted IQS can accomplish the Area Under Curve (AUC) value more than 0.96 when the threshold is set to 0.5, and more than 0.80 when threshold is 0.9. As [9] suggested previously, the imputation accuracy may overestimate the quality of imputation. The results shown in Figure 4 and 5 show that the predicted IQS performs better than the predicted imputation accuracy with a larger AUC in all four scenarios, suggesting that the predicted IQS can filter out more presumed false positives than the predicted imputation accuracy, and the results are consistent in all four scenarios. We also show the curves of the true imputation accuracy as a reference.

## Conclusion

We propose a *ν*-SVR based approach to the estimation of the true IQS of imputations of SNPs with unknown true genotypes. We show that our regression model generalizes equally well across SNP selections by different platforms and across different ethnic groups and disease populations. The model performed particularly well for predicting the true chance agreement of imputation. We also showed that the estimated IQS can be used to filter false positive associations in a GWAS to some extent. The results suggest that it is feasible to apply a regression model to predict the true IQS.

Our future work includes an effort to extend the feature set to improve the regression performance for predicting *P_o _*and the IQS and to pool together a wide variety of data sets including different SNPs and populations as the training examples so that one model can be used to estimate the IQS for all imputations. When the model is sufficiently robust, our long-term goal is to impute to the same size all genotype data in repositories of GWAS data (to as large as the most advanced platforms) and apply this regression model to attach an estimated IQS to all imputations in addition to the posteriori probability from the imputation program and make the results available in the public domain.

## Competing interests

The authors declare that they have no competing interests.

## Authors' contributions

YHH and CNH developed methods and designed the experiments. YHH and CNH drafted the manuscript. JPR, SFS, JLA, YA and JAT participated in the design of the study. JLA and JAT helped to revise the manuscript. CNH was responsible for all aspects of the project.

## References

[B1] Howie BN Donnelly P Marchini J A flexible and accurate genotype imputation method for the next generation of genome-wide association studies PLoS Genet 2009 5 6 e1000529 10.1371/journal.pgen.1000529 19543373 PMC2689936

[B2] Browning SR Missing data imputation and haplotype phase inference for genome-wide association studies Human Genetics 2008 124 5 439 450 10.1007/s00439-008-0568-7 18850115 PMC2731769

[B3] Browning BL Yu Z Simultaneous genotype calling and haplotype phasing improves genotype accuracy and reduces false-positive associations for genome-wide association studies The American Journal of Human Genetics 2009 85 6 847 861 10.1016/j.ajhg.2009.11.004 PMC2790566 19931040

[B4] Browning BL Browning SR A unified approach to genotype imputation and haplotype-phase inference for large data sets of trios and unrelated individuals The American Journal of Human Genetics 2009 84 2 210 223 10.1016/j.ajhg.2009.01.005 PMC2668004 19200528

[B5] Browning SR Browning BL Rapid and accurate haplotype phasing and missing-data inference for whole-genome association studies by use of localized haplotype clustering The American Journal of Human Genetics 2007 81 5 1084 1097 10.1086/521987 PMC2265661 17924348

[B6] Browning SR Browning BL High-resolution detection of identity by descent in individuals The American Journal of Human Genetics 2010 86 4 526 539 10.1016/j.ajhg.2010.02.021 PMC2850444 20303063

[B7] Browning BL Browning SR Efficient multilocus association testing for whole genome association studies using localized haplotype clustering Genetic Epidemiology 2007 31 5 365 375 10.1002/gepi.20216 17326099

[B8] Li Y Abecasis aR Gonc Mach 1.0: rapid haplotype reconstruction and missing genotype inference American Journal of Human Genetic 2006 S79 S79 2290

[B9] Lin P Hartz SM Zhang Z Saccone SF Wang J Tischfield JA Edenberg HJ Kramer JR MGoate A Bierut LJ Rice JP for the COGA Collaborators COGEND Collaborators G A New statistic to evaluate imputation reliability PLoS ONE 2010 5 e9697 10.1371/journal.pone.0009697 20300623 PMC2837741

[B10] Cohen J A coefficient of agreement for nominal scales Educational and Psychological Measurement 1960 20 37 46 10.1177/001316446002000104

[B11] Smola AJ Schölkopf B A tutorial on support vector regression Statistics and Computing 2004 14 3 199 222

[B12] Cortes C Vapnik V Support-vector networks 1995 20 3 273 297

[B13] Chen P Lin CJ Schölkopf B A tutorial on *ν*-support vector machines 2003

[B14] Chang CC Lin CJ Training nu-support vector regression theory and algorithms 10.1162/089976602760128081 12180409

[B15] Schölkopf B Smola AJ Williamson RC Bartlett PL New support vector algorithms 2000 10.1162/089976600300015565 10905814

[B16] Chang CC Lin CJ LIBSVM a library for support vector machines ACM Transactions on Intelligent Systems and Technologies 2011 2 1 27

[B17] Consortium TIH The International HapMap project Nature 2003 426 789 796 10.1038/nature02168 14685227

[B18] May C Slingsby M Jeffreys A Human recombination hotspots: before and after the HapMap Project 2008 2 195 244

[B19] Broet P Camilleri-Broet S Zhang S Alifano M Bangarusamy D Battistella M Wu Y Tuefferd M Regnard JF Lim E Tan P Miller LD Prediction of clinical outcome in multiple lung cancer cohorts by integrative genomics: implications for chemotherapy selection Cancer Res 2009 69 3 1055 1062 10.1158/0008-5472.CAN-08-1116 19176396

[B20] Peng CH Liao CT Peng SC Chen YJ Cheng AJ Juang JL Tsai CY Chen TC Chuang YJ Tang CY Hsieh WP Yen TC A novel molecular signature identified by systems genetics approach predicts prognosis in oral squamous cell carcinoma PLoS ONE 2011 6 8 e23452 10.1371/journal.pone.0023452 21853135 PMC3154947

[B21] Barrett T Troup DB Wilhite SE Ledoux P Evangelista C Kim IF Tomashevsky M Marshall KA Phillippy KH Sherman PM Muertter RN Holko M Ayanbule O Yefanov A Soboleva A NCBI GEO: archive for functional genomics data sets 10 years on Nucleic Acids Research 2011 39 suppl 1 D1005 D1010 21097893 10.1093/nar/gkq1184 PMC3013736

[B22] Baum LE Petrie T Statistical inference for probabilistic functions of finite state Markov Chains The Annals of Mathematical Statistics 1966 37 6 1554 1563 10.1214/aoms/1177699147

[B23] Nothnagel M Ellinghaus D Schreiber S Krawczak M Franke A A comprehensive evaluation of SNP genotype imputation Human Genetics 2009 125 2 163 171 10.1007/s00439-008-0606-5 19089453

